# EGF regulation of proximal tubule cell proliferation and VEGF‐A secretion

**DOI:** 10.14814/phy2.13453

**Published:** 2017-09-28

**Authors:** Diana Zepeda‐Orozco, Hsiang M. Wen, Bradley A. Hamilton, Nandita S. Raikwar, Christie P. Thomas

**Affiliations:** ^1^ Division of Pediatric Nephrology Stead Family Department of Pediatrics, Dialysis and Transplantation University of Iowa Carver College of Medicine Iowa City Iowa; ^2^ Department of Internal Medicine University of Iowa Carver College of Medicine Iowa City Iowa; ^3^ VA Medical Center Iowa City Iowa

**Keywords:** EGF, HIF‐1 *α*, proliferation, proximal tubule cells, VEGF‐A

## Abstract

Proximal tubule cell (PTC) proliferation is critical for tubular regeneration and recovery from acute kidney injury. Epidermal growth factor (EGF) and vascular endothelial growth factor (VEGF‐A) are important for the maintenance of tubulointerstitial integrity and can stimulate PTC proliferation. We utilized HK‐2 cells, an immortalized human PTC line, to characterize the EGF‐dependent regulation of VEGF‐A secretion and proliferation in PTCs. We demonstrate that EGF stimulates VEGF‐A secretion via the EGF receptor (EGFR) and stimulates cell proliferation via activation of the VEGF receptor, VEGFR‐2. EGFR activation promotes MAPK (ERK1/2) activation and HIF‐1*α* expression, which are required for basal and EGF‐stimulated VEGF‐A secretion. EGF also stimulates the phosphorylation of P70S6 kinase (P70S6K), the downstream target of mTORC1. Rapamycin decreased basal and EGF stimulated HIF‐1*α* and enhanced MAPK (ERK1/2) activation, while MAPK (ERK/12) inhibition downregulated HIF‐1*α* expression and the phosphorylation of p70S6K. EGF stimulation of p70S6K was also independent of p‐AKT. Inhibition of the mTORC1 pathway with rapamycin abolished phosphorylation of p70S6K but had no effect on VEGF‐A secretion, indicating that EGF‐stimulated VEGF‐A secretion did not require mTORC1 pathway activation. We demonstrate evidence of a complex crosstalk between the MAPK/ERK and mTORC1 pathways, wherein MAPK (ERK1/2) activation stimulates p‐P70S6K, while p‐P70S6K activation seems to inhibit MAPK (ERK1/2) in EGF‐treated HK‐2 cells. Our results suggest that EGF stimulates MAPK (ERK1/2) in HK‐2 cells, which in turn increases HIF‐1*α* expression and VEGF‐A secretion, indicating that VEGF‐A mediates EGF‐stimulated cell proliferation as an autocrine proximal tubular epithelial cell growth factor.

## Introduction

Developing and adult kidneys undergo continuous segment‐specific tubulogenesis highlighting the importance of tubular epithelial cell proliferation in maintaining overall kidney health (Rinkevich et al. 2014). Following acute kidney injury (AKI), proximal tubule cells (PTCs) produce a number of growth factors that regulates their proliferation (Basile et al. 2012), which is important for tubular regeneration and renal recovery following AKI (Humphreys et al. 2008; Kramann et al. 2015; Lombardi et al. 2016). Therefore, it is essential to understand the mechanisms regulating PTCs response to proliferative cues in order to improve therapeutic approaches to AKI and the progression of chronic kidney disease. There is ample evidence supporting important roles for vascular endothelial growth factor‐A (VEGF‐A) and epidermal growth factor (EGF) in the maintenance of tubulointerstitial integrity and the response to AKI (Schrijvers et al. 2004; Maeshima et al. 2006; Melenhorst et al. 2008; Zeng et al. 2009; Doi et al. 2010; Staruschenko et al. 2013; Tang et al. 2013).

The VEGF family currently comprises seven members: VEGF‐A, ‐B, ‐C, ‐D, ‐E, ‐F, and placenta growth factor (Hoeben et al. 2004). VEGF‐A is expressed in human and rodent kidneys predominantly in glomerular podocytes and tubular epithelial cells (Kanellis et al. 2000b; Hoeben et al. 2004; Schrijvers et al. 2004; Doi et al. 2010; Dimke et al. 2015). VEGF‐A expression is primarily regulated by the hypoxia‐inducible protein complex HIF‐1, which is a heterodimer consisting of the HIF‐1*α* and HIF‐1*β* subunits (Hoeben et al. 2004). HIF‐1*α* is degraded under normoxic conditions by ubiquitination. In a hypoxic environment HIF‐1*α* is stabilized and its ubiquitination is inhibited, which leads to an increase in HIF‐1*α* expression and transcriptional activation of target genes such as VEGF‐A (Yee Koh et al. 2008; Gunaratnam and Bonventre 2009). Additionally, growth factors have been shown to increase HIF‐1*α* and VEGF‐A expression (Hoeben et al. 2004; Yee Koh et al. 2008). In particular, EGF, transforming growth factor β (TGF‐*β*), and platelet‐derived growth factor (PDGF) are known to increase VEGF‐A expression (Hoeben et al. 2004; Schrijvers et al. 2004). There are two VEGF‐A receptors: the fms‐like tyrosine kinase Flt‐1 (VEGFR‐1) and the kinase domain region Flk‐1 (VEGFR‐2/KDR) (Hoeben et al. 2004; Larsen et al. 2011). VEGFR‐1 and VEGFR‐2 are predominantly expressed in glomerular and peritubular endothelial cells, but expression has also been demonstrated in tubular epithelial cells (Kanellis et al. 2000a; Schrijvers et al. 2004). In vitro studies in different PTC lines show that VEGF‐A signals via phosphorylation of VEGFR‐2 and not VEGFR‐1 and induces cell proliferation (Kanellis et al. 2000a; Villegas et al. 2005). More importantly, specific changes in tubular VEGF‐A alter the tubulointerstitium in vivo. Transgenic mice with overexpression of VEGF‐A within renal tubules show an increase in tubular epithelial cell growth and cyst formation, increased proliferation of peritubular capillaries and fibroblasts, and glomerulomegaly (Hakroush et al. 2009). On the other hand, VEGF‐A knockdown in tubular cells produces smaller kidneys with reduced peritubular capillaries and increased renal EPO production and polycythemia, presumably from renal interstitial ischemia (Dimke et al. 2015).

EGF and epithelial growth factor receptor (EGFR) signaling have an important role in renal organogenesis, electrolyte homeostasis, renal metabolism, AKI, and chronic kidney disease (Melenhorst et al. 2008; Zeng et al. 2009; Tang et al. 2013). The EGFR family, also known as ErbB receptors, includes EGFR (HER1, ErbB1), HER2/neu (ErbB2), HER3 (ErbB3), and HER4 (ErbB4). EGFR ligands include EGF, TGF‐*α*, heparin‐binding EGF‐like growth factor (HB‐EGF), amphiregulin, betacellulin, epigen, and epiregulin (Melenhorst et al. 2008; Zeng et al. 2009). Depending on the ErbB ligand and the composition of receptor dimers, a variety of downstream signaling transduction pathways can be activated by EGF stimulation, including (1) the mitogen‐activated protein kinase (MAPK/ERK) pathway via activation of Raf‐1, MAPK/ERK kinase (MEK)1/2, and MAPK(ERK1/2); and (2) class I phosphatidylinositol 3‐kinase (PI3K), via phosphorylation of AKT‐protein kinase B (AKT) (Melenhorst et al. 2008). Both the PI3K/AKT and MAPK/ERK pathways are able to activate the mTORC1 pathway by inhibition of the tuberous sclerosis complex 1 and complex 2 (TSC1/2), leading to phosphorylation of S6 kinase 1 (P70S6K) (Julien et al. 2010; Chen et al. 2015). In the kidney, even though EGF is strongly expressed in PTCs in vivo, and EGFR is expressed in every nephron segment, their expression can change dramatically during renal development and pathological conditions (Staruschenko et al. 2013). EGFR signaling induces PTC proliferation and differentiation in renal development and following AKI (Maeshima et al. 2006; Melenhorst et al. 2008; Zeng et al. 2009; Tang et al. 2013).

Despite the importance of EGF and VEGF‐A in proximal tubule proliferation and their implications for maintenance of the tubulointerstitium, our understanding of the relationship between both growth factors in PTCs is incomplete. In this study, we examined the effects of EGF in renal PTC proliferation and VEGF‐A secretion using immortalized human PTCs (HK‐2 cells). Our results show how EGF regulates VEGF‐A secretion by HK‐2 cells and its effects on PTC proliferation. We also demonstrate, for the first time, that regulation of VEGF‐A in PTCs is orchestrated by the complex cross‐link between MAPK/ERK and mTORC1 pathways via regulation of HIF‐1*α*. These results are of importance because they demonstrate that when we target mTORC1/P70S6K and MAPK/ERK signaling cascades there are compensatory mechanisms within PTCs. Additionally, changes in growth factor expression or responsiveness could contribute to the increased susceptibility to tissue injury, vascular rarefaction, and progressive fibrosis.

## Materials and Methods

### Cell culture

Human kidney proximal tubule HK‐2 (ATCC, CRL‐2190) cells were grown in Keratinocyte Serum Free Media (K‐SFM) supplemented with 0.05 mg/mL bovine pituitary extract (BPE) and 5 ng/mL Epidermal Growth Factor (EGF) provided with the K‐SFM kit (Gibco 10724‐011, Thermo Fisher Scientific, Waltham, MA). Cells were maintained in a humidified 5% CO_2_ atmosphere at 37°C.

### Cell treatments

For all experiments, HK‐2 cells were initially incubated in K‐SFM media without BPE and EGF for 20–24 h. Then cells were placed in fresh K‐SFM and subjected to specified treatments for 6, 18, or 24 h as indicated in the figure legends. EGF (PromoKine C‐6017) was used at 1, 5, or 50 ng/mL. MAPK/ERK inhibitor U0126 (Promega V112A, Madison, WI) was used at 10 *μ*mol/L. Rapamycin (Thermo Fisher Scientific, Cat#: BP29631) was used at 50 nmol/L. The epidermal growth factor receptor (EGFR) inhibitor, gefitinib (Santa Cruz sc‐202166, Dallas TX), was used at 0.1 *μ*mol/L, 0.5 *μ*mol/L, or 1 *μ*mol/L. The EGFR inhibitor erlotinib hydrochloride (Sigma‐Aldrich CDS022564), was used at 1 *μ*mol/L; the VEGFR‐2 kinase inhibitor VII, SKLB 1002 (Calbiochem 676505, Millipore Sigma, Billerica, MA) was used at 5 *μ*mol/L or 10 *μ*mol/L.

### HIF‐1 α SiRNA transfection

At 70% confluence, HK‐2 cells were transiently transfected with 23 nmol/L HIF‐1*α* siRNA (Santa Cruz, sc‐44225) or scrambled siRNA (Santa Cruz, sc‐37007) using Lipofectamine RNAiMAX™ Transfection Reagent (Life Technologies, 13778080, Thermo Fisher Scientific) according to the manufacturer's protocol. Forty‐eight hours after transfection, cells were incubated in K‐SFM media without BPE or EGF for 20 h, and then cells were subjected to specific treatment for 6 h.

### Cell lysates

Cells were rinsed with ice‐cold phosphate‐buffered solution and lysed in RIPA Buffer (Pierce 89901, Thermo Fisher Scientific) containing EDTA‐free protease inhibitors (Roche 11836170001, Indianapolis, IN) and Phosphatase Inhibitor Cocktail Set II (Calbiochem, 524625, Millipore Sigma). Cell lysates were vortexed at 4°C for 30 min and centrifuged at 12,000*g*, 4°C for 20 min. Supernatants were transferred to fresh tubes. Protein concentration was determined by BCA Protein Assay (Pierce 23227, Thermo Fisher Scientific).

### Western blot analysis

Equal amounts of protein were resolved on sodium dodecyl sulfate polyacrylamide gel electrophoresis (SDS‐PAGE gels) and transferred to polyvinylidene difluoride (PVDF) membranes using the Bio‐Rad Trans‐Blot Turbo™ Transfer System (Hercules, CA). Membranes were blocked for 30 min with 5% nonfat dry milk, and a primary antibody was applied for 2 h at room temperature or overnight at 4°C. The following primary antibodies were used: rabbit anti‐HIF‐1*α* (Abcam ab2185, Cambridge, MA) at 1:1,000 dilution; rabbit anti‐phospho‐p44/42 MAPK (pERK1/2) (Cell Signaling, CS9101L, Danvers, MA) at 1:1,000; rabbit anti‐p44/42 MAPK (ERK1/2) (Cell Signaling, 4695) at 1:1000; rabbit anti‐phospho‐AKT (S473) (Cell Signaling, CS9271L) at 1:1000; rabbit anti‐AKT (Cell Signaling, 9292) at 1:1000; rabbit anti‐phospho‐p70S6K (Thr421/Ser424, Cell Signaling, CS9204L) at 1:250; rabbit anti‐p70S6K (Cell Signaling, 9202) at 1:1000; rabbit anti‐phospho‐EGFR (Tyr1068, Cell signaling, CS2234) at 1:250; and mouse anti‐tubulin (Sigma Aldrich, T5168, St, Louis, MO) at 1:1,000. Blots were washed three times with Tris‐buffered saline containing 0.1% Triton X‐100, then incubated with peroxidase‐conjugated secondary antibody (goat anti‐rabbit or goat anti‐mouse, 1:10,000, Jackson Laboratories, West Grove, PA) for 1 hour at room temperature, followed by three more washes as described above. Proteins were visualized with ECL SuperSignal™ West Maximum Sensitivity Substrate (Thermo Fisher Scientific 34096), according to the provided protocol; signals were captured using VisionWorks™ LS image acquisition software and the EC3 Imaging system from UVP LLC (Upland, CA). Quantification of western blot band density was performed using ImageJ.

### Human VEGF immunoassay

A quantikine human VEGF‐A enzyme‐linked immunosorbent assay (ELISA) kit was purchased from R&D System (DVE00, Minneapolis, MN). Cell culture media were collected immediately after cell treatments. Particulates were removed by centrifugation. Samples were stored at −20°C until ELISA was performed according to the manufacturer's protocol.

### Proliferation assay

A BrdU Cell Proliferation Assay kit was purchased from Millipore (2752, Millipore Sigma). Cells were seeded at a density of 20,000 per well in a 96‐well plate in K‐SFM media without BPE or EGF. BrdU was added 3 h prior to the end of the cell treatment period. Drug concentration and cell treatment durations are indicated in the figure legends. Assays were performed according to the manufacturer's protocol. Briefly, a mouse monoclonal antibody was used to detect BrdU followed by the addition of a peroxidase‐conjugated goat anti‐mouse antibody. A peroxidase substrate was used to develop a color reaction, and absorbance was measured using a FLUOstar OPTIMA microplate reader (BMG Labtech, Cary, NC) at wavelength 450 nmol/L.

### Cell count

Cells were seeded in a six‐well plate at 1.5 × 105 cells per well. The next day, cells were purged with K‐SFM media without BPE or EGF for 24 h. Then cells were subject to treatment for 24 h as indicated in the figure legends. Samples were counted using a Countess Automated Cell Counter (Thermofisher).

## Results

### EGF stimulates HK‐2 cell VEGF‐A secretion via the EGF receptor, and stimulates HK‐2 cell proliferation via activation of VEGFR‐2

**Figure 1:**
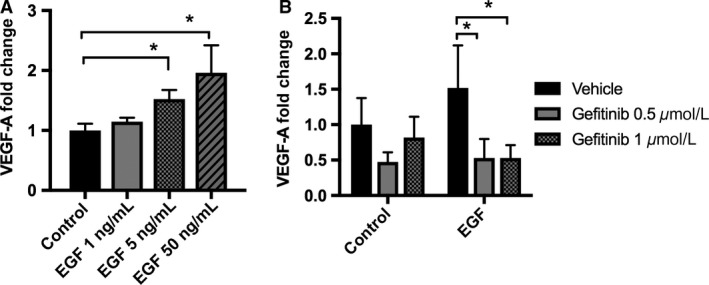
Epithelial growth factor (EGF) stimulates vascular endothelial growth factor‐A (VEGF‐A) secretion in human kidney cells (HK‐2) via stimulation of EGF receptor (EGFR). VEGF‐A concentration was measured by enzyme‐linked immunosorbent assay (ELISA) in supernatants from HK‐2 cells. Cells were grown to 70% confluence on K‐SFM with 5 ng/mL of EGF, and 0.05 mg/mL of BPE. Prior to experiments, HK‐2 cell media was replaced by K‐SFM without BPE or EGF for 20 h. (A) Cells were subjected to 1, 5, and 50 ng/mL of EGF in fresh K‐SFM for 6 h to assess dose response. Data are expressed as mean fold change compared to control (K‐SFM only) ± SD (*n* = 3–7). *P < 0.05 Control versus EGF by one‐way ANOVA. (B) Cells were incubated in K‐SFM with or without 5 ng/mL of EGF and were treated with EGFR inhibitor gefitinib at 0.5 *μ*mol/L or 1 *μ*mol/L or vehicle for 6 hr. Data are expressed as mean fold change compared to control (K‐SFM only) ± SD (*n* = 3–4). *P < 0.05 vehicle versus gefitinib and EGF versus control by two‐way ANOVA.

**Figure 2:**
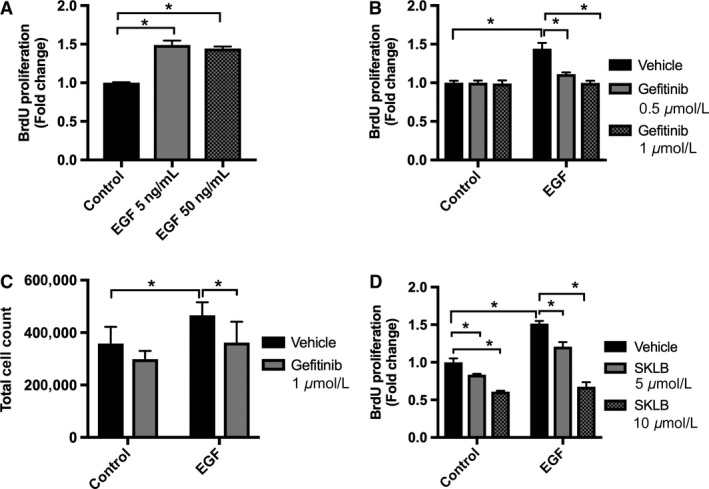
EGF dependent HK‐2 cell proliferation is inhibited by blockade of EGFR with gefitinib, and blockade of vascular endothelial growth factor receptor‐2 (VEGFR‐2) with SKLB. Measurement of HK‐2 cell proliferation was performed using a BrdU cell proliferation assay kit as detailed in methods. Cells were seeded in a 96‐well plate in K‐SFM media without EGF or BPE for 20 h, then media was replaced with fresh K‐SFM supplemented with different experimental conditions. (A) Cells were treated for 6 h with EGF 5 or 50 ng/mL or vehicle. Data are expressed as fold change from control condition ± SD (*n* = 3). *P < 0.05 control versus EGF with one‐way ANOVA. (B) HK‐2 cells were incubated in K‐SFM with or without 5 ng/mL of EGF and were treated with EGFR inhibitor gefitinib at 0.5 *μ*mol/L or 1 *μ*mol/L or vehicle for 18 h. Data are expressed as fold change from control condition ± SD (*n* = 3). *P < 0.05 vehicle versus gefitinib, EGF versus control with two‐way ANOVA. (C) HK‐2 cells were incubated in K‐SFM with and without 5 ng/mL of EGF for 24 h. Cells were quantified using a Countess Automated Cell Counter. Data are expressed as total cell count ± SD (*n* = 5–6). *P < 0.05 vehicle versus gefinitib with two‐way ANOVA. (D) HK‐2 cells were incubated in K‐SFM with and without 5 ng/mL of EGF for 24 h, followed by treatment with SKLB or vehicle for 6 h. Data are expressed as fold change from control condition ± SD (*n* = 3). *P < 0.05 vehicle versus SKLB and EGF versus control with two‐way ANOVA.

VEGF‐A is an important growth factor for PTCs, and in these cells EGFR activation has been previously implicated in the stimulation of VEGF‐A expression and secretion (Rudnicki et al. 2009; Fernandez‐Martinez and Lucio‐Cazana 2015). However, the signaling mechanism involved in EGF‐mediated VEGF‐A secretion in PTCs is unknown. We first sought to determine the effect of EGFR blockade on VEGF‐A secretion. Cells grown in K‐SFM showed a significant dose‐dependent EGF stimulation of VEGF‐A secretion at 5 ng/mL and 50 ng/mL (Fig. 1A). Gefitinib, an EGFR blocker, abolished VEGF‐A secretion by EGF‐stimulated HK‐2 cells, demonstrating that EGF simulates VEGF‐A secretion via the EGFR in HK‐2 cells (Fig. 1B). We then examined the relationship between EGF and VEGF‐A in the regulation of PTC proliferation, as both EGF and VEGF have been shown to upregulate PTC proliferation in vivo and in vitro (Norman et al. 1987; Kanellis et al. 2000a; Zhuang et al. 2004; Villegas et al. 2005; Chen et al. 2012). HK‐2 cells treated with 5 and 50 ng/mL of EGF had a significant increase in cell proliferation, which was inhibited by the EGFR blocker gefitinib (Fig. 2A, B, and C). We confirmed our results by measuring proliferation in the presence of a second EGFR blocker, erlotinib, in HK‐2 cells stimulated with EGF (Fig. S1). We then tested if the effect of EGF on PTC proliferation required VEGF‐A secretion and the activation of its receptor. HK‐2 cells treated with EGF in the presence of the VEGFR‐2 inhibitor SKLB showed a significant reduction in proliferation, suggesting that EGF stimulates HK‐2 cell proliferation by upregulation of VEGF‐A secretion and subsequent signaling via VEGFR‐2 (Fig. 2D).

### EGFR activation upregulates HIF‐1α, p‐MAPK(ERK1/2), and p‐P70S6K expression in HK‐2 cells

**Figure 3:**
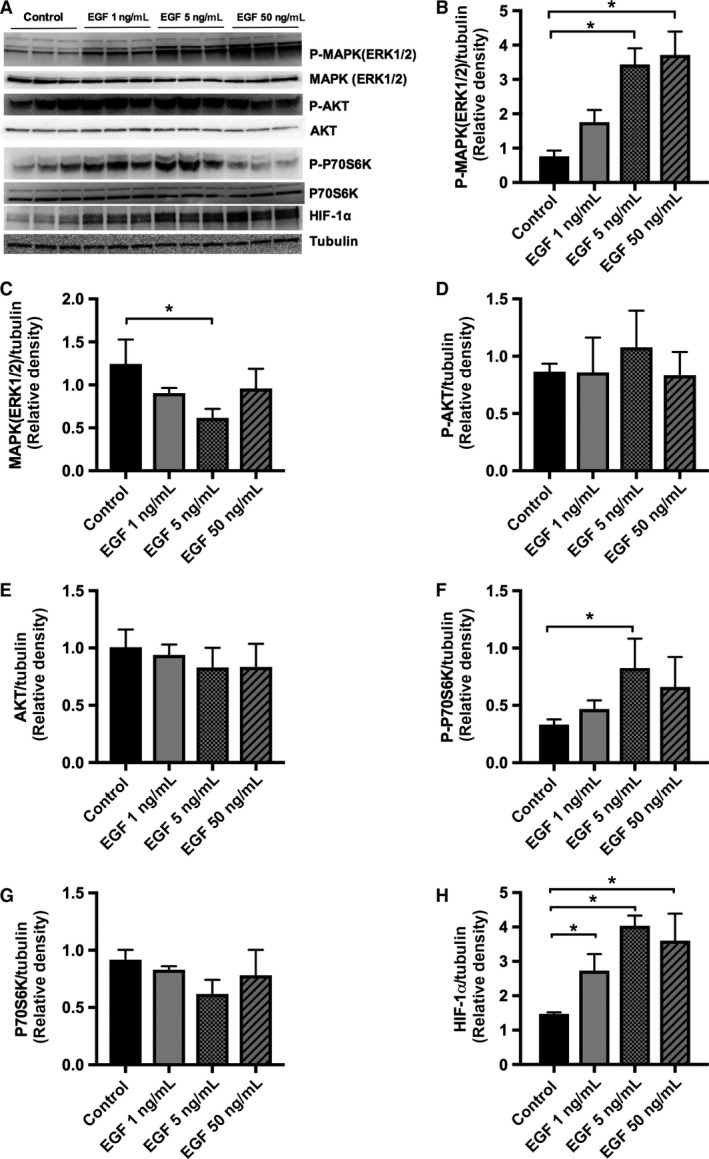
Effect of EGF treatment in HK‐2 cell expression of *P*‐MAPK (ERK1/2), P‐P70S6K, and HIF‐1*α*. Cells were grown to 70% confluence in K‐SFM with 5 ng/mL of EGF, and 0.05 mg/mL of BPE. Twenty hours prior to experiment, HK‐2 cell media was replaced by K‐SFM without BPE or EGF. Cells were then subjected to EGF 1, 5, and 50 ng/mL of EGF in fresh K‐SFM for 6 h. Cell lysate was immunoblotted with anti‐phospho‐44/42 MAPK (ERK1/2), anti 44/42 MAPK (ERK1/2), anti‐phospho‐AKT, anti‐AKT, anti‐HIF‐1*α*, anti‐phospho‐P70S6K antibodies; tubulin was used as loading control. (A) Representative blot is shown. Quantification of western blot band density ratio of (B) P‐MAPK(ERK1/2), (C) MAPK (ERK1/2), (D) P‐AKT, (E) AKT, (F) P‐P70S6K, (G) P70S6K, and (H) HIF‐1*α* to loading control was performed using ImageJ. Data are expressed as mean ± SD (*n* = 3). *P < 0.05 control versus EGF with one‐way ANOVA.

EGFR activation is known to stimulate MAPK/ERK and PI3K/AKT signaling cascades in PTCs (Zhuang et al. 2004; Lee et al. 2007; Chen et al. 2012). Both the EGFR‐mediated PI3K/AKT and MAPK/ERK stimulation have been implicated in the mTORC1‐dependent phosphorylation of P70S6K in mammary epithelial cells via inhibition of TSC1/2 (Galbaugh et al. 2006; Julien et al. 2010). However, the role of these signaling pathways in EGF‐stimulated VEGF‐A secretion and PTC proliferation is not well known. We first evaluated, by immunoblotting, the effects of EGF on phosphorylation of MAPK (ERK1/2) and AKT in HK‐2 cells, and we demonstrated that EGF significantly upregulated p‐MAPK(ERK1/2) and had no significant effect on p‐AKT, suggesting that EGFR signaling was primarily via MAPK(ERK1/2) pathway (Fig. 3A, B and D). There was downregulation of total MAPK (ERK1/2) at 5 ng/mL, but no significant change in total MAPK (ERK1/2) expression at 1 ng/mL or 50 ng/mL, nor in total AKT at any concentration (Fig. 3A, C and E). Interestingly, phosphorylation of P70S6K was increased with addition of 5 ng/mL of EGF, but not with 50 ng/mL EGF (Fig. 3A and F), with no significant change in total P70S6K expression at any concentration (Fig. 3A and G). These results suggest that the stimulation of p‐P70S6K is either biphasic or accompanied by a negative feedback loop on the mTORC1 pathway. Lastly, EGFR activation upregulated HIF‐1*α* dose‐dependently in HK‐2 cells (Fig. 3A and H). The effects of EGF in HIF‐1*α* under normoxic conditions has been previously described in tumor cells (Peng et al. 2006; Lee et al. 2009; Larsen et al. 2011), but has never been shown in PTC line before.

### EGF stimulates VEGF‐A secretion via MAPK(ERK1/2) signaling and HIF‐1α

**Figure 4:**
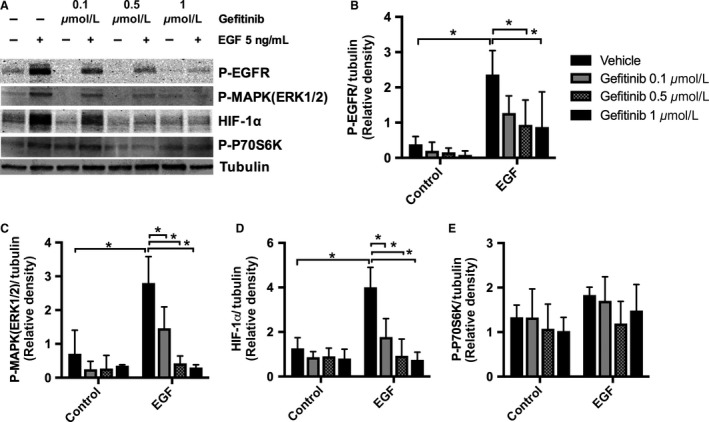
Effect of EGFR blockade with gefitinib in HK‐2 cell expression of P‐MAPK (ERK1/2), P‐70S6K, and HIF‐1*α*. Cells were grown to 70% confluence in K‐SFM with 5 ng/mL of EGF, and 0.05 mg/mL of BPE. Twenty hours prior to experiments, HK‐2 cell media was replaced by K‐SFM without BPE or EGF for 20 h. HK‐2 cells were incubated in K‐SFM with or without 5 ng/mL of EGF and were treated with EGFR inhibitor gefitinib at 0.1 *μ*mol/L, 0.5 *μ*mol/L, 1 *μ*mol/L or vehicle for 6 h. (A) Representative blot. Quantification of western blot band density ratio of (B) P‐EGFR, (C) P‐MAPK, (D) HIF‐1*α*, and (E) P‐P70S6K to loading control was performed using ImageJ. Data are expressed as mean ± SD (*n* = 3–5). *P < 0.05 vehicle versus gefitinib and EGF versus control with two‐way ANOVA.

**Figure 5:**
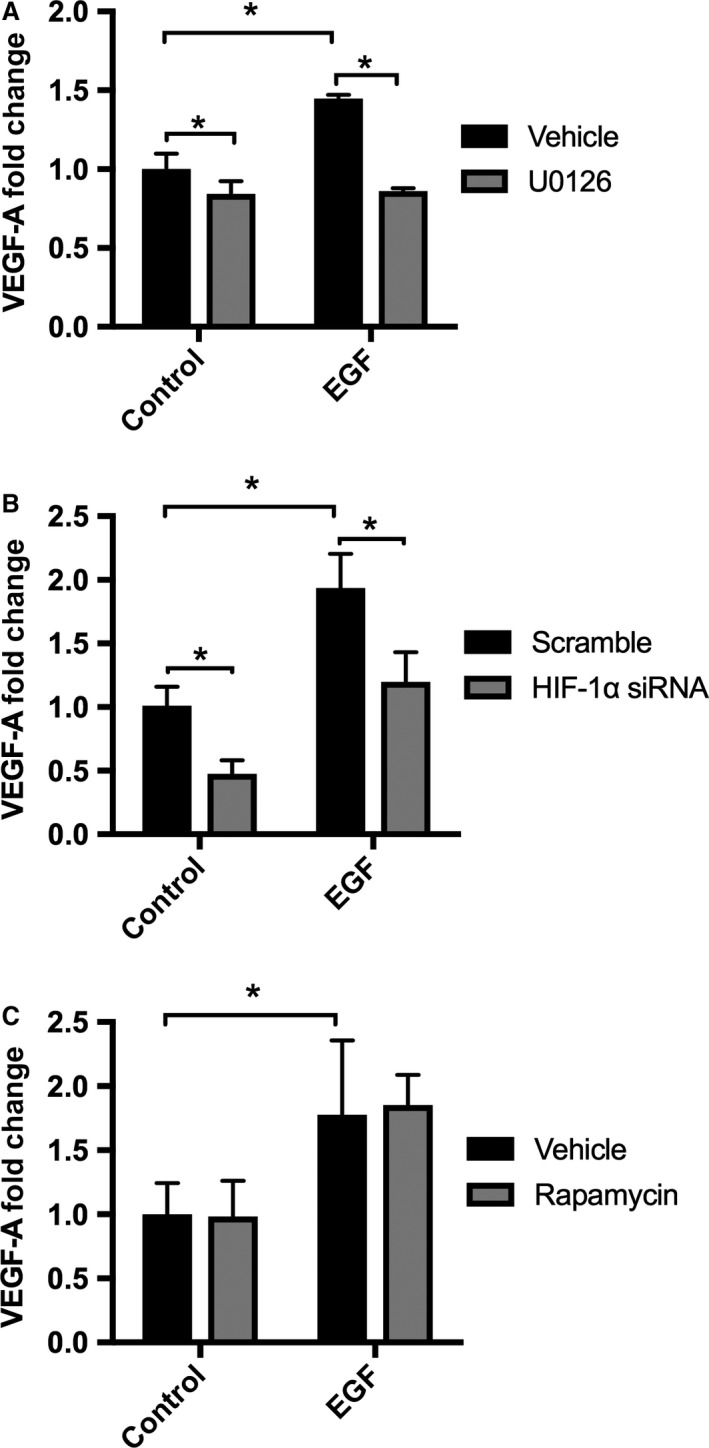
VEGF‐A secretion by HK‐2 cells after blockade of MAPK (ERK1/2) signaling with UO126, mTOR inhibition with rapamycin, and HIF‐1*α* knockdown by siRNA. VEGF‐A concentration was measured by enzyme‐linked immunosorbent assay (ELISA) in supernatant from HK‐2 cells. Cells were grown to 70% confluence on K‐SFM with 5 ng/mL of EGF, and 0.05 mg/mL of BPE. Twenty hours prior to experiments, HK‐2 cell media was replaced by K‐SFM without BPE or EGF. Cells were then incubated in K‐SFM with or without 5 ng/mL of EGF and were treated with (A) MAPK/ERK inhibitor U0126 at 10 *μ*mol/L. (B) At 70% confluence, HK‐2 cells were transfected with 23 nmol/L of HIF‐1*α* siRNA or scramble siRNA. Forty‐eight hours after transfection, cells were treated with K‐SFM media without BPE or EGF for 20 h, and then cells were subjected to 5 ng/mL of EGF or vehicle for additional 6 h. (C) Cells were grown to 70% confluence on K‐SFM with 5 ng/mL of EGF, and 0.05 mg/mL of BPE. Twenty hours prior to experiments, HK‐2 cell media was replaced by K‐SFM without BPE or EGF. Cells were then incubated in K‐SFM with or without 5 ng/mL of EGF and were treated with rapamycin at 50 nmol/L, or vehicle. Data are expressed as mean fold change compared to control condition (K‐SFM only) ± SD. *P < 0.05 vehicle versus UO126, (*n* = 3–6) or rapamycin (*n* = 6–10), or HIF‐1*α* siRNA (*n* = 6) and EGF versus control by two‐way ANOVA.

To determine the contribution of these second messenger systems in the regulation of EGF‐dependent VEGF‐A secretion by HK‐2 cells, we used the EGFR blocker gefitinib to evaluate downstream signaling pathways by immunoblotting (Fig. 4A). Gefitinib decreased EGFR phosphorylation (Fig. 4A and B) and downregulated EGF‐stimulated p‐MAPK (ERK1/2) and HIF‐1*α* expression in HK‐2 cells (Fig. 4A, C and D). However, there was no significant change in expression of p‐P70S6K (Fig. 4A and E). Using erlotinib, a second EGFR inhibitor, we confirmed that EGFR blockade significantly downregulates p‐MAPK (ERK1/2) and had no effect on p‐AKT phosphorylation (FigS2. A–D). Our results suggested that EGF may stimulate VEGF‐A secretion in HK‐2 cells by activation of the MAPK/ERK pathway, and by increasing expression of mTORC1 and HIF‐1*α* but not by activation of the PI3K/AKT pathway. To verify the direct effects of HIF‐1*α*, MAPK/ERK, and mTORC1 pathways on HK‐2 cell VEGF‐A secretion, we tested the effects of the MAPK/ERK kinase blockade (UO126), mTORC1 inhibition (rapamycin), and HIF‐1*α* knockdown on EGF‐stimulated HK‐2 cells. Both MAPK/ERK kinase inhibition and HIF‐1*α* knockdown under normoxic conditions reduced EGF‐dependent VEGF‐A secretion in HK‐2 cells (Fig. 5A and B) confirming that EGF‐mediated stimulation of EGFR regulates VEGF‐A secretion in HK‐2 cells via MAPK (ERK1/2) signaling and the transcription factor HIF‐1*α*. In contrast, rapamycin had no effect on VEGF‐A secretion, suggesting that EGFR stimulated VEGF‐A via a direct effect of MAPK (ERK1/2) and the regulation of HIF‐1*α*, but independent of the mTORC1 pathway (Fig. 5C).

### Crosstalk between MAPK/ERK and mTORC1 pathways in EGF‐stimulated HK‐2 cells

**Figure 6:**
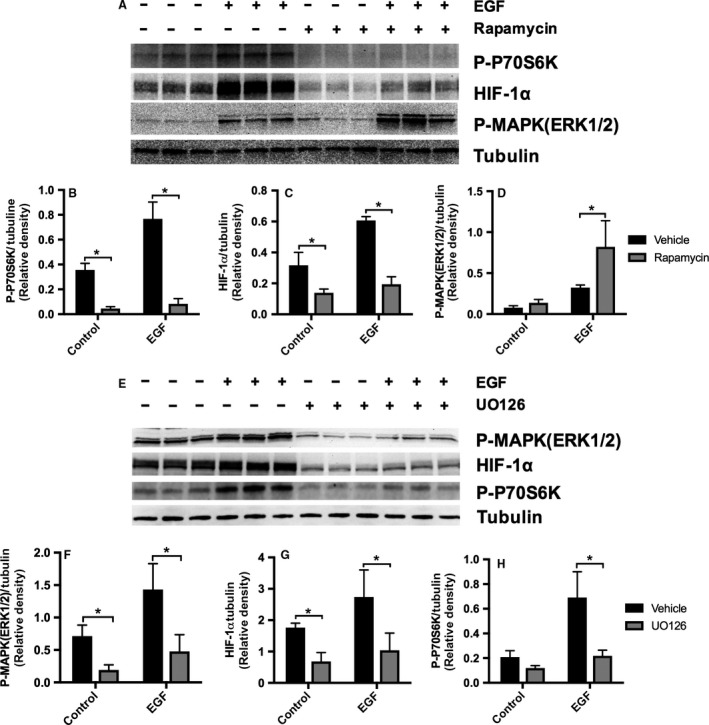
Crosstalk between mTOR and MAPK/ERK pathways shown by expression of P‐MAPK (ERK1/2), P‐P70S6K, and HIF‐1*α* following mTOR and MAPK/ERK inhibition in EGF‐stimulated HK‐2 cells. HK‐2 cells were incubated in K‐SFM with or without 5 ng/mL of EGF and treated with mTOR inhibitor rapamycin at 50 nmol/L, or MAPK/ERK inhibitor U0126 at 10 *μ*mol/L, or vehicle for 6 h. Cell lysate was immunoblotted with anti‐phospho‐44/42 MAPK (ERK1/2), anti‐HIF‐1*α*, and anti‐phospho‐P70S6K antibodies; tubulin was used as loading control. Representative western blot of (A) mTOR inhibition and quantification of western blot band density ratio of (B) P‐70S6K, (C) HIF‐1*α*, and (D) P‐MAPK (ERK1/2) to loading control was performed using ImageJ. Data are expressed as mean ± SD (*n* = 3–6). *P < 0.05 vehicle versus rapamycin and EGF versus control (*n* = 3–6) by two‐way ANOVA. Representative western blot of (E) MAPK(ERK1/2) inhibition and quantification of western blot band density ratio of (F) P‐MAPK (ERK1/2), (G) HIF‐1*α*, and (H) P‐70S6K, to loading control was performed using ImageJ. Data are expressed as mean ± SD (*n* = 3–6). *P < 0.05 vehicle versus UO126 and EGF versus control (*n* = 3–6) by two‐way ANOVA.

**Figure 7:**
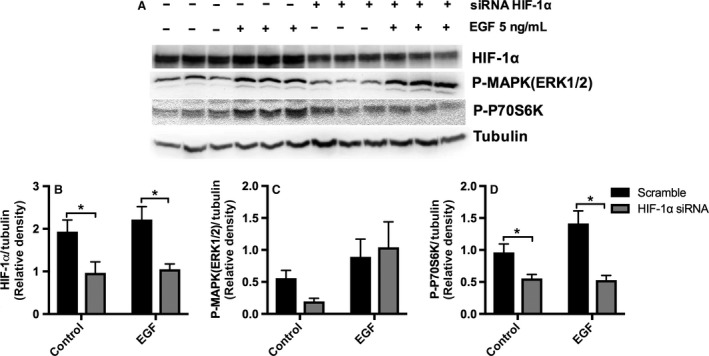
Effects of HIF‐1*α* knockdown on P‐MAPK (ERK1/2), and P‐P70S6K in EGF‐stimulated HK‐2 cells. At 70% confluence, HK‐2 cells were transiently transfected with HIF‐1*α* siRNA or scrambled siRNA. Forty‐eight hours after transfection, cells were incubated in K‐SFM media without BPE or EGF for 20 h, and then cells were subjected to specific treatment for 6 h. Cell lysate was immunoblotted with anti‐phospho‐44/42 MAPK (Erk1/2), anti‐HIF‐1*α*, and anti‐phospho‐P70S6K antibodies; tubulin was used as loading control. (A) Representative western blot and quantification of western blot band density ratio of (B) HIF‐1*α*, (C) P‐MAPK(ERK1/2), and (D) P‐70S6K to tubulin loading control using ImageJ. Data are expressed as mean ± SD (*n* = 3). *P < 0.05 scramble versus HIF‐1*α* siRNA by two‐way ANOVA.

Previous studies have implicated HIF‐1*α* and both the PI3K/AKT/mTORC1 and MAPK/ERK pathways in VEGF‐A expression in PTCs with hypoxia and hypoxia‐reperfusion injury (Hellwig‐Burgel et al. 2005; Conde et al. 2012). Our results showed that EGF stimulated both MAPK/ERK and mTORC1 pathways, but had no effect on the PI3K/AKT pathway, suggesting that mTORC1 stimulation in EGF‐treated cells was regulated primarily by the MAPK(ERK1/2) signaling cascade and was independent of the PI3K/AKT pathway. The EGF‐dependent VEGF‐A secretion by HK2 cells was inhibited by HIF‐1*α* knockdown and MAPK (ERK1/2) blockade but not by mTORC1 inhibition, confirming that EGF‐mediated VEGF‐A secretion is regulated by MAPK (ERK1/2) and HIF‐1*α* and not by mTORC1. Even though EGF stimulated phosphorylation of P70S6K (Fig. 3D), our results demonstrated that the stimulation of p‐P70S6K had no significant effects on VEGF‐A secretion, and the bimodal expression of p‐P70S6K suggested a possible feedback loop regulating MAPK‐dependent stimulation of p‐P70S6K. To characterize a possible crosstalk between the mTORC1 pathway and MAPK (ERK1/2) pathways, we tested the effect of rapamycin in HK‐2 cells (Fig. 6A). Rapamycin downregulated the phosphorylation of P70S6K and decreased the basal and EGF‐stimulated expression of HIF‐1*α* (Fig. 6A, B and C). However, rapamycin had no effect on basal p‐MAPK (ERK1/2) and enhanced rather than inhibited the EGF stimulation of p‐MAPK (ERK1/2) (Fig. 6A and D). On the other hand, the MAPK/ERK inhibitor UO126 effectively blocked MAPK (ERK1/2) activation and downregulated HIF‐1*α* expression, and the phosphorylation of P70S6K (Fig. 6A, E and H). Together, our results suggest that EGF stimulates MAPK (ERK1/2) in HK‐2 cells, which in turn increases p‐P70S6K, HIF‐1*α* expression, and VEGF‐A secretion. HIF‐1*α* may be necessary but not sufficient to regulate the MAPK (ERK1/2) effect on VEGF‐A secretion because rapamycin does not affect VEGF‐A secretion despite HIF‐1*α* inhibition, suggesting that an alternative MAPK (ERK1/2)‐dependent but HIF‐1*α*‐independent pathway is also involved in regulation of VEGF‐A secretion by PTCs. In addition, there is evidence of a crosstalk between MAPK (ERK1/2) and mTORC1 pathways; MAPK (ERK1/2) activation stimulates p‐P70S6K, while P70S6K activation seems to inhibit p‐MAPK(ERK1/2) in EGF‐treated HK‐2 cells. We then evaluated the effects of HIF‐1*α* knockdown in HK‐2 cells using HIF‐1*α* siRNA (Fig. 7A–D). HIF‐1*α* knockdown significantly downregulated HIF‐1*α* (Fig. 7B) and p‐P70S6K (Fig. 7D) in control and EGF‐stimulated cells. There was a trend toward upregulation of p‐MAPK (ERK1/2) expression; however, it did not reach statistical significance (Fig. 7C).

## Discussion

Following AKI, surviving tubular epithelial cells have the ability to proliferate in order to replace tubular epithelial cells that have been lost (Humphreys et al. 2008). Isolated PTC injury can also drive capillary rarefaction, the initiation of interstitial fibrosis, and the development of glomerulosclerosis, perhaps when the ability to proliferate and repair injured epithelium is compromised (Grgic et al. 2012). EGF and VEGF‐A are important in PTC proliferation and in maintenance of tubulointerstitial integrity (Norman et al. 1987, 1990; Nowak and Schnellmann 1995; Kanellis et al. 2000a; Zhuang et al. 2004; Villegas et al. 2005; Hakroush et al. 2009; Chen et al. 2012; Dimke et al. 2015). Treatments with VEGF‐A and EGF have been shown to be protective in animal models of AKI (Humes et al. 1989; Norman et al. 1990; Leonard et al. 2008). Although EGF has been reported to stimulate VEGF‐A mRNA expression in PTCs (Rudnicki et al. 2009), the mechanisms involved in that regulation were unknown and the role of VEGF‐A in mediating EGF‐dependent cell proliferation was also unknown. Here, we show that EGF stimulates HK‐2 cell VEGF‐A secretion by activation of the MAPK (ERK1/2) signaling pathway and stimulation of HIF‐1*α* transcription factor, VEGF‐A, which then binds to VEGFR‐2 and stimulates PTC proliferation.

The relationship between EGF and VEGF‐A was previously described in cancer cells where it was noticed that EGF treatment increases VEGF‐A, and EGFR blockade had the opposite response (Goldman et al. 1993; Petit et al. 1997; Ciardiello et al. 2001; Pore et al. 2006). In our experiments, we sought to determine the molecular mechanisms involved in the regulation of this interaction in HK‐2 cells. Consistent with our results, EGFR transactivation by prostaglandins is known to induce MAPK (ERK1/2)‐dependent VEGF‐A secretion (Fernandez‐Martinez and Lucio‐Cazana 2015). In vitro studies have shown that mechanical injury of PTCs led to EGFR activation and stimulated the phosphorylation of AKT and MAPK (ERK1/2), and that addition of exogenous EGF enhanced the response. Additionally, EGFR inhibition blocked phosphorylation of MAPK (ERK1/2) and AKT, and PI3K inhibition downregulated PTC proliferation (Zhuang et al. 2004). We evaluated the effects of mTOR activation in our model because in previous studies in PTCs undergoing hypoxia‐reoxygenation injury, mTOR signaling measured by p‐AKT and p‐P70S6K appeared to regulate VEGF‐A expression via HIF‐1*α* (Conde et al. 2012). We demonstrated that HK‐2 cells treated with EGF had significant upregulation of p‐P70S6K and activation of HIF‐1*α*. However, phosphorylation of P70S6K had a dose‐dependent, biphasic pattern. Rapamycin, an mTORC1 inhibitor, did not affect HK‐2 cell VEGF‐A secretion despite the inhibition of P70S6K phosphorylation and the downregulation of HIF‐1*α*. Although EGF treatment stimulated the mTORC1 pathway in HK‐2 cells, the effects appeared to be independent of p‐AKT signaling, and there appeared to be a negative feedback mechanism triggered by mTORC1 activation. In contrast, HK‐2 cells treated with an EGFR blocker had significant downregulation of p‐MAPK (ERK1/2), and when MAPK (ERK1/2) phosphorylation was pharmacologically blocked with UO126, there was significant downregulation of both p‐P70S6K and HIF‐1*α* expression, as well as VEGF‐A secretion. Our experiments showed that MAPK (ERK1/2) signaling is the principal stimulatory pathway in EGF‐mediated VEGF‐A secretion by HK‐2 cells, without involvement of p‐AKT or p‐P70S6K in regulation of VEGF‐A secretion. Growth factors can signal to mTOR either through PI3K/AKT or via MAPK (ERK1/2)‐mediated phosphorylation of TSC2 (Ma et al. 2005; Mendoza et al. 2011; Zoncu et al. 2011; Fruman and Rommel 2014), and mTORC1 inhibition can actually block the P70S6K‐mediated feedback loop and upregulate the PI3K and MAPK/ERK signaling cascade (Carracedo et al. 2008; Zoncu et al. 2011). We evaluated if the mTORC1 resistance to EGF stimulation of VEGF‐A secretion in HK‐2 cells was explained by interactions between MAPK/ERK and mTORC1 pathways. We found that rapamycin increased phosphorylation of MAPK (ERK1/2) in EGF‐stimulated HK‐2 cells, and UO126 inhibited phosphorylation of both, MAPK (ERK1/2) and P70S6K. We did not see an effect of rapamycin on AKT phosphorylation (Fig. S3). These findings suggested to us that EGF‐mediated stimulation of mTORC1 pathway is via MAPK (ERK1/2) signaling, and that EGFR stimulation triggers a negative feedback loop orchestrated by mTORC1, which may prevent excessive secretion of VEGF‐A secretion in PTCs.

EGF‐treated HK‐2 cells had significant upregulation of the transcription factor HIF‐1*α* under normoxic conditions. Currently, it is recognized that HIF‐1*α* is not only a regulator of oxygen homeostasis, angiogenesis, and erythropoiesis, but it also has an important role in glucose metabolism, cellular proliferation, and apoptosis (Semenza 2001; Schofield and Ratcliffe 2004). In PTCs, HIF‐1*α* has been involved in regulation of hypoxia‐induced cell responses including VEGF‐A synthesis, glucose consumption, major histocompatibility complex class I‐related chain expression, in vitro epithelial‐to‐mesenchymal transition, renal fibrosis, and apoptosis (Biju et al. 2005; Hellwig‐Burgel et al. 2005; Sun et al. 2009; Luo et al. 2010; Conde et al. 2012). It has been proposed that HIF‐1*α* expression is downregulated by medications like metformin via reduction in oxygen consumption rate in PTCs (Takiyama et al. 2011). We showed that HK‐2 cells treated with EGF have significant dose‐dependent upregulation of HIF‐1*α*. Both MAPK/ERK and mTOR pathways signal via HIF‐1*α* in EGF‐stimulated HK‐2 cells, as the inhibition of either significantly downregulated HIF‐1*α* expression. We showed that HIF‐1*α* gene knockdown decreases VEGF‐A secretion by HK‐2 cells. Our findings are consistent with previous reports showing that EGFR transactivation by prostaglandin E2 results in MAPK (ERK1/2)‐dependent upregulation of HIF‐1*α*, as well as in VEGF‐A production (Fernandez‐Martinez and Lucio‐Cazana 2015). However, HK‐2 cells transfected with HIF‐1*α* siRNA had downregulation of VEGF‐A secretion not only in EGF‐treated HK‐2 cells, but also under basal conditions, suggesting that HIF‐1*α* regulates basal and EGF‐dependent VEGF‐A secretion. Additionally, rapamycin significantly downregulated HIF‐1*α* but had no effect on VEGF‐A secretion, suggesting that MAPK (ERK1/2) regulates HIF‐1*α*‐dependent and HIF‐1*α*‐independent stimulation of VEGF‐A secretion. Possible transcription factors involved in EGFR/MAPK(ERK1/2)‐dependent regulation of VEGF‐A secretion include STAT3 and Sp1(Larsen et al. 2011); however, the study of the HIF‐1*α*‐independent regulation of VEGF‐A is beyond the scope of our study. The crosstalk between MAPK (ERK1/2) and mTORC1 signaling could orchestrate HIF‐1*α*‐dependent tubular epithelial cell proliferation and changes in growth and metabolism (Chen et al. 2015). Finally, It has been shown that VEGF‐A increased PTC proliferation via VEGFR‐2 activation (Kanellis et al. 2000a; Villegas et al. 2005). HK‐2 cells treated with EGF had a significant increase in their proliferation, which was inhibited by EGFR and VEGFR‐2 blockade, suggesting that EGF‐dependent HK‐2 cell proliferation is mediated by an autocrine effect of VEGF‐A signaling via VEGFR‐2.

**Figure 8:**
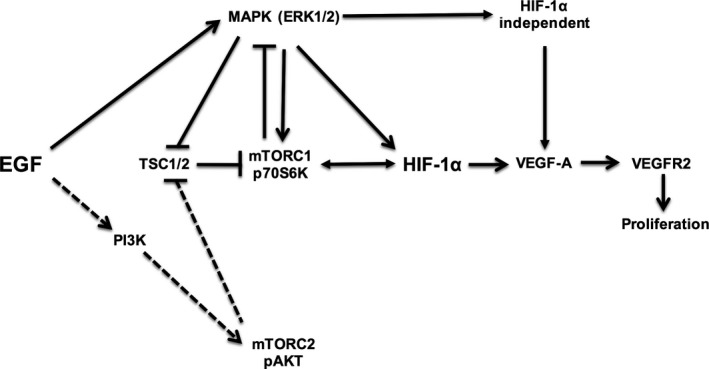
Schematic representation of EGF‐mediated regulation of proximal tubule cell VEGF‐A secretion and proliferation. Bold lines represent EGF‐dependent regulation of VEGF‐A. The dotted lines represent previously described pathways involved in EGF signaling, but this was not found in our model.

VEGF‐A and EGF are important growth factors involved in the regulation of PTC proliferation. We demonstrated that EGF stimulates VEGF‐A secretion and HK‐2 cell proliferation via complex interactions between MAPK/ERK and mTORC1 pathways, which then signal to the transcription factor HIF‐1*α* (Fig. 8). Because PTC proliferation is important for the response to kidney injury, our results highlight critical pathways within PTCs that could be manipulated to regulate proliferation in vitro and in vivo.

## Conflict of Interest

The authors declare no conflicts of interest.

## Data Accessibility

## Supporting information




**Figure S1.** EGF‐dependent HK‐2 cell proliferation is inhibited by blockade of EGFR with second EGFR blocker erlotinib.

**Figure S2**. Effect of EGFR blockade with erlotinib in HK‐2 cell expression of P‐MAPK (ERK1/2), and P‐AKT.

**Figure S3.** Effects of rapamycin on P‐AKT.
