# Supplementary figures and images for: EGF regulation of proximal tubule cell proliferation and VEGF‐A secretion
Source: Physiol Rep. 2017 Sep 28;5(18):e13453. doi: 10.14814/phy2.13453 (PMC5617933; doi:10.14814/phy2.13453)

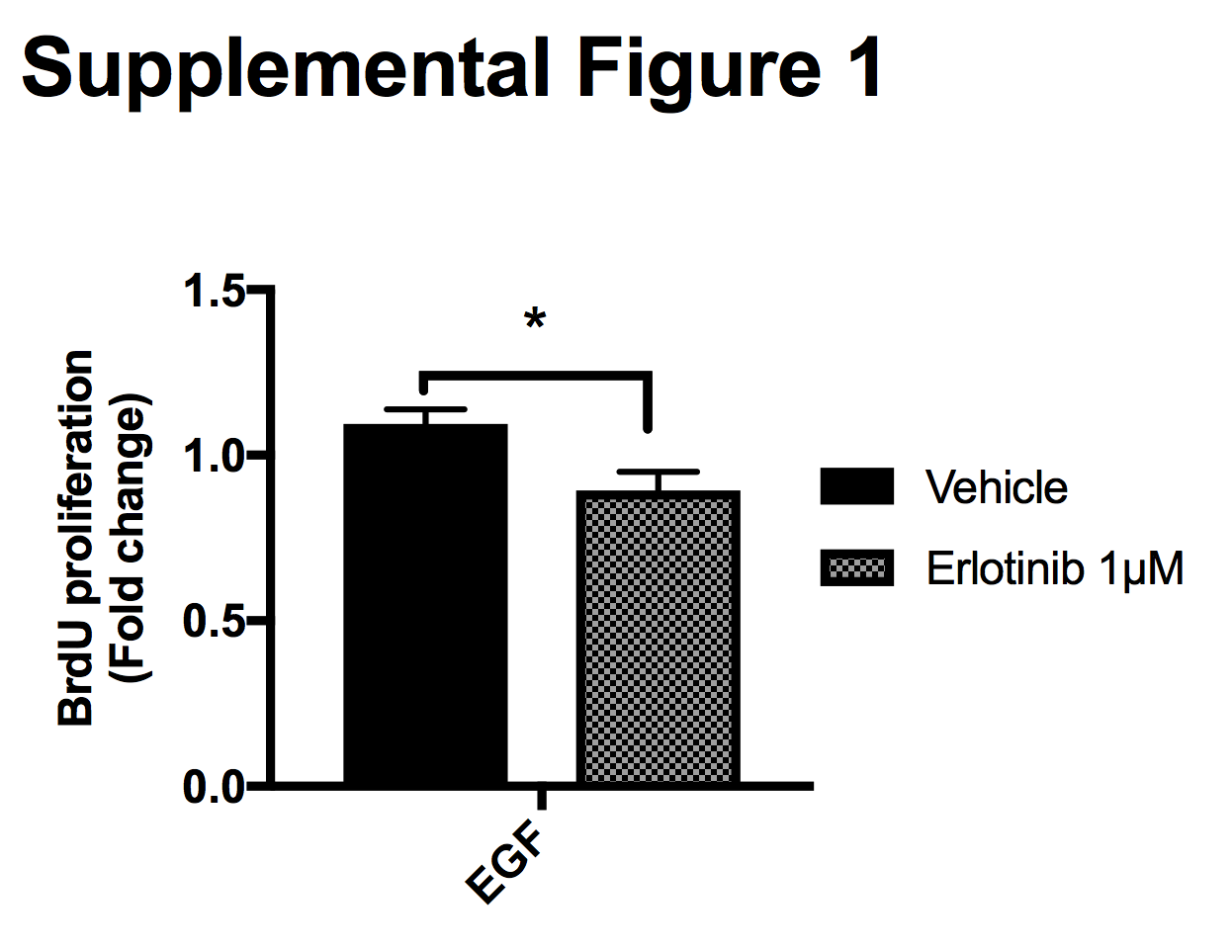

Supplement: Supplementary file 1 — Figure S1. EGF‐dependent HK‐2 cell proliferation is inhibited by blockade of EGFR with second EGFR blocker erlotinib. [file PHY2-5-e13453-s001.tiff]

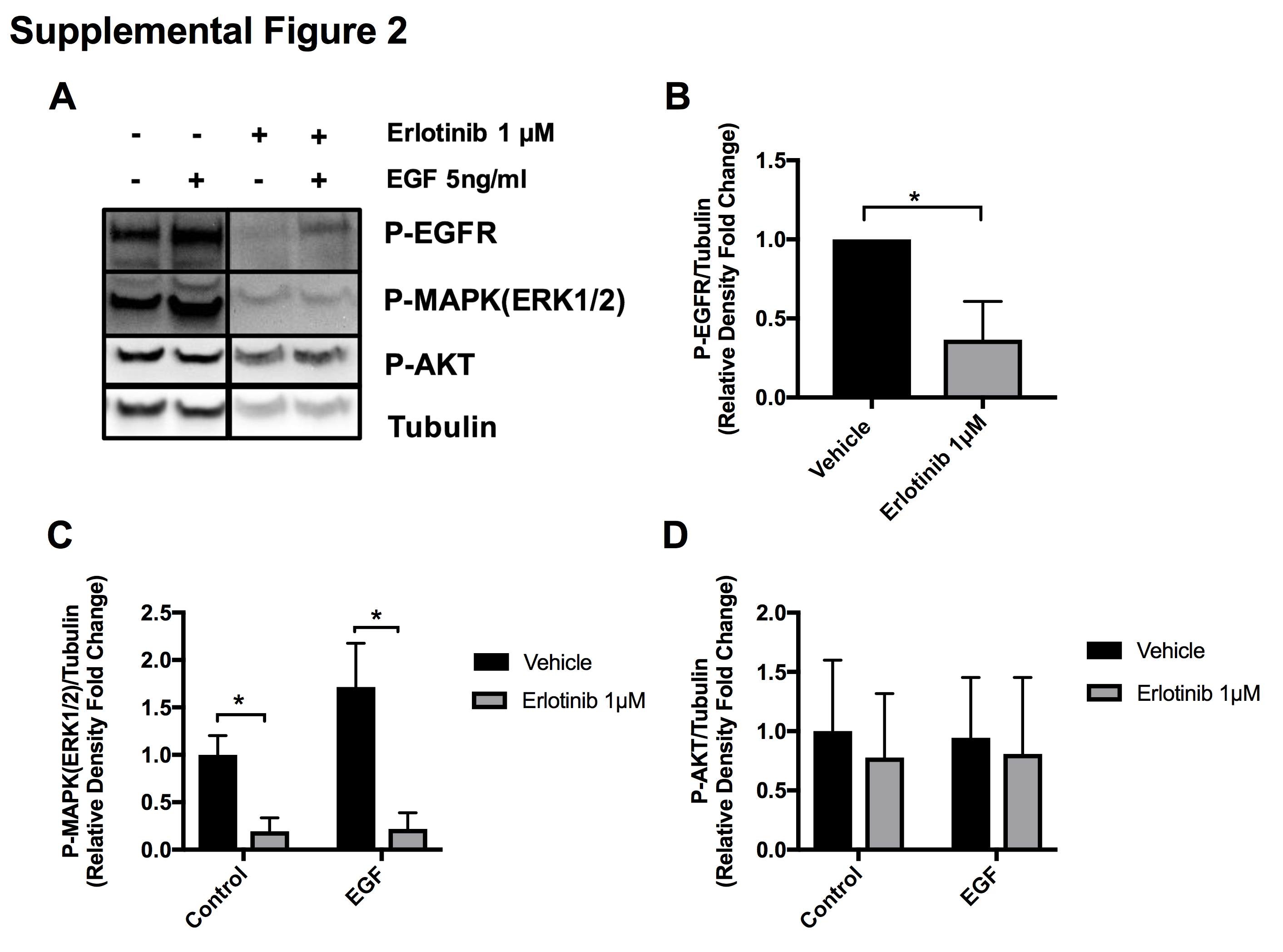

Supplement: Supplementary file 2 — Figure S2. Effect of EGFR blockade with erlotinib in HK‐2 cell expression of P‐MAPK (ERK1/2), and P‐AKT. [file PHY2-5-e13453-s002.tiff]

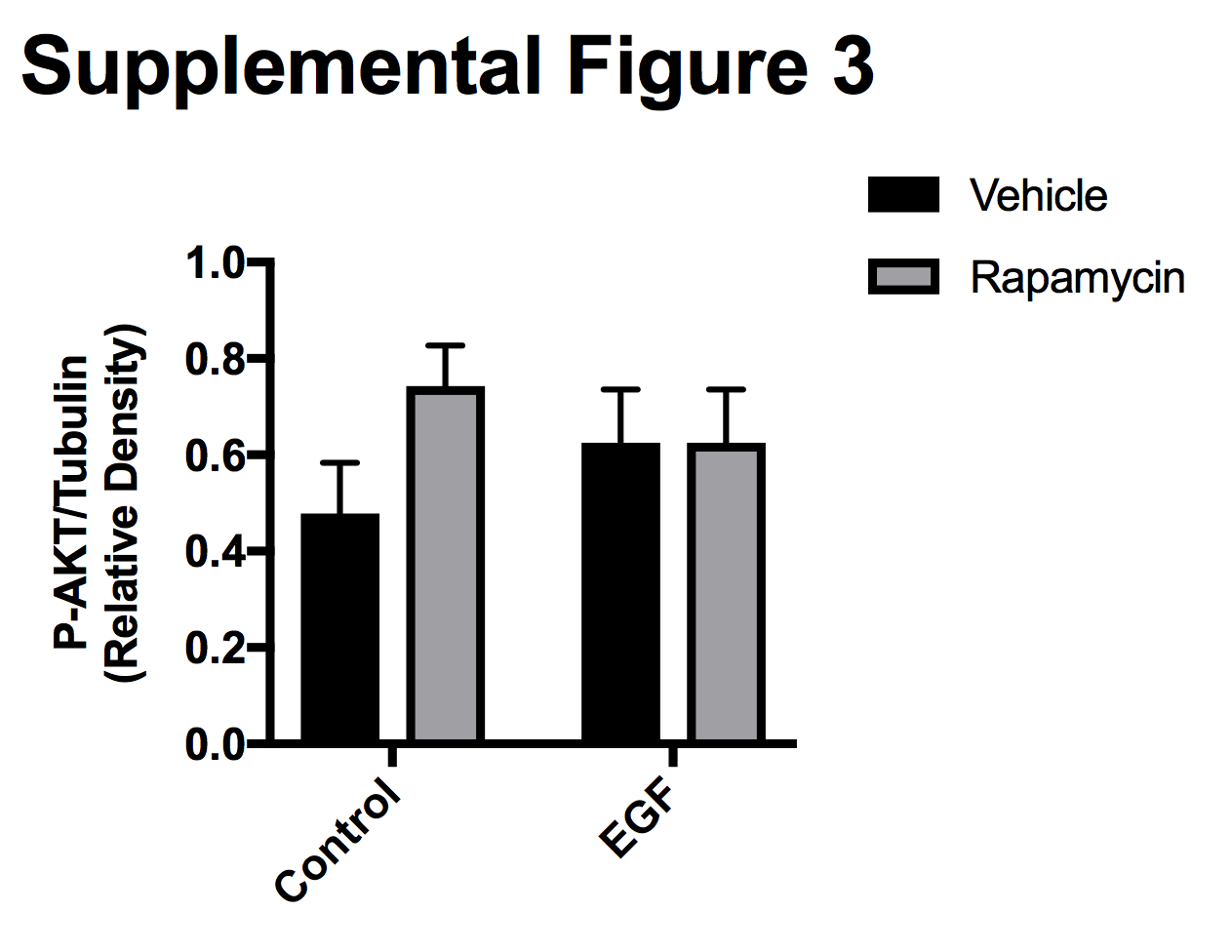

Supplement: Supplementary file 3 — Figure S3. Effects of rapamycin on P‐AKT. [file PHY2-5-e13453-s003.tiff]
